# Rational transplant timing and dose of mesenchymal stromal cells in patients with acute myocardial infarction: a meta-analysis of randomized controlled trials

**DOI:** 10.1186/s13287-016-0450-9

**Published:** 2017-01-28

**Authors:** Zi Wang, Lingling Wang, Xuan Su, Jun Pu, Meng Jiang, Ben He

**Affiliations:** Department of Cardiology, Renji Hospital, School of Medicine, Shanghai Jiaotong University, 160 Pujian Road, Shanghai, 200127 China

**Keywords:** Mesenchymal stromal cell, Acute myocardial infarction, Transplantation timing, Cell dose

## Abstract

**Background:**

Mesenchymal stromal cells (MSCs) are considered to have a modest benefit on left ventricular ejection fraction (LVEF) in patients with acute myocardial infarction (AMI). However, the optimal injection timing and dose needed to induce beneficial cardiac effects are unknown. The purpose of this meta-analysis was to identify an optimal MSC transplantation time and cell dose in the setting of AMI to achieve better clinical endpoints.

**Methods:**

The authors conducted a systematic review of studies published up to June 2016 by searching PubMed, EMBASE, MEDLINE, and the Cochrane Library for relevant randomized controlled trials (RCTs).

**Results:**

Eight prospective RCTs with 449 participants were included. The pooled results revealed that patients in the MSC group had no significant increase in LVEF from baseline compared with that in the control group (1.47% increase, 95% confidence interval (CI) −4.5 to 7.45; *I* ^2^ = 97%; *P* > 0.05). A subgroup analysis was conducted to explore the results according to differences in transplantation time and dose of MSCs injected. For transplantation timing, the LVEF of patients accepting a MSC infusion within 1 week was significantly increased by 3.22% (95% CI 1.31 to 5.14; *I* ^2^ = 0; *P* < 0.05), but this increase was insignificant in the group that accepted an MSC infusion after 1 week (−0.35% in LVEF, 95% CI −10.22 to 9.52; *I* ^2^ = 99%; *P* > 0.05). Furthermore, patients accepting a MSC dose of less than 10^7^ cells exhibited an LVEF improvement of 2.25% compared with the control (95% CI 0.56 to 3.93; *I* ^2^ = 9%; *P* < 0.05). Combining transplantation time and cell dose indicates that a significant improvement of LVEF of 3.32% was achieved in the group of patients injected with <10^7^ MSCs within 1 week (95% CI 1.14 to 5.50; *I* ^2^ = 0; *P* = 0.003).

**Conclusions:**

Transplantation time and injected cell dose are key factors that determine the therapeutic effect of stem cell therapy. The injection of no more than 10^7^ MSCs within 1 week for AMI after percutaneous coronary intervention might improve left ventricular systolic function. Further studies on the mechanism and the effectiveness of MSCs for long-term therapy are warranted.

## Background

Stem cell transplantation in the setting of acute myocardial infarction (AMI) has been implemented for decades [1, 2]. Although early revascularization can save part of the ischemic myocardium, non-renewable necrotic myocardial cells call for stem cell therapy to provide changes in reducing mortality rate and improving quality of life [3, 4]. Mesenchymal stromal cells (MSCs) serve as a major cell candidate and a promising stem cell type to improve cardiac function following transplantation to infarcted myocardium [5, 6].

Chen et al. were the first to report that MSC transplantation in AMI patients could increase left ventricular ejection fraction (LVEF) by 12% [7]. The results prompted an increase in the numbers of centers that use the therapeutic potential and safety of MSCs in AMI in the context of regenerative therapy and immune modulation [8, 9]. However, most of the reported studies were small or had conflicting outcomes [10–12].

Bone marrow stem cell transfer is an emerging therapy for AMI patients. Several meta-analyses have indicated the effectiveness and safety of bone marrow stem cells in improving LVEF, but no consistent results are available regarding the optimal transplantation time, and no data on dosage have been obtained [13–17]. The above meta-analyses [13, 16, 17] included trials using hybrid cells as the study group, which might hide the effectiveness of certain stem cell types. Considering that varying the time interval from stem cell harvest to reinfusion may influence cytoactivity and the cell dosage may affect myocardial recovery, the authors conducted a meta-analysis of current randomized controlled trials (RCTs) using MSCs for AMI to investigate the effectiveness of MSC transplantation in AMI patients and to study the effects of transplantation time and transplant dose of MSCs; the results obtained might be helpful for the design of more precise clinical studies.

## Methods

### Review questions

This systematic review was conducted according to the methods recommended by published guidelines for meta-analyses [4]. The authors attempted to address the following questions: within which time period and using which cell dose will MSC transplantation provide the most benefit for AMI patients?

### Data source and search strategy

Relevant studies were identified by searching PubMed, MEDLINE, the Cochrane Library (to June 2016), and internet-based sources of information on cardiology. The following search terms were used, alone or in combination [18]: *mesenchymal stromal cells, mesenchymal stem cells, stem cells, stem cell, stromal cells, stromal cell, acute myocardial infarction, myocardial infarction, coronary artery disease, cardiac repair*. No language limit was applied. The search was limited to RCTs and the transplantation of MSCs only in the test group.

### Eligibility criteria

Inclusion criteria for the randomized AMI groups in the present analysis were as follows: (1) RCTs; (2) transplanted stem cells were limited to the MSC cell type, but the cell dose or administration route were unrestricted; (3) studies that were conducted in patients with ST-segment or non-ST-segment elevation myocardial infarction treated with primary percutaneous coronary intervention (PCI) after AMI; (4) studies that used cell-based therapy within 3 months after PCI in AMI patients; (5) studies that involved participants receiving standard therapy in both cell groups and control arms while the control arm did not receive stem cells; and (6) studies that did not restrict MSC resources in terms of origin (human bone marrow, human umbilical cords, or adipose tissue) but that did not use granulocyte colony-stimulating factor (G-CSF).

### Data extraction and quality assessment

The authors analyzed the reports published for each trial, and standard information was entered into a spreadsheet. Two investigators independently extracted data from the studies, including publication date, study design, sample size, method used to assess myocardial function, patient baseline characteristics, cell resource and dose, delivery route, primary intervention, baseline LVEF, time from intervention to cell infusion, cardiac medication, follow-up duration, and change in LVEF, left ventricular end-systolic volume (LVESV), and left ventricular end-diastolic volume (LVEDV) outcomes. Echocardiography, single-photon emission computed tomography, positron emission computed tomography, and cardiac magnetic resonance imaging (MRI) functional data were available; echocardiography data were preferentially used unless MRI data were available [19–21]. In case of missing or unclear data for the primary or secondary endpoints, at least two separate attempts were made at least 3 weeks apart to clarify the data by contacting the primary authors. When any disagreement occurred between the investigators, a third reviewer independently adjudicated based on the data.

### Outcomes

The primary endpoints were to find the optimal injected dose and its timing to induce better LVEF from baseline to follow-up.

### Quality assessment

Study quality was assessed based on the use of appropriate randomization, concealment of treatment allocation, similarity of treatment groups at baseline, the provision of a description of the eligibility criteria, completeness of the follow-up duration, and the use of an intention-to-treat analysis. The quality of the studies was assessed in accordance with the Jüni criteria [22], using a risk scale to evaluate the quality as high, low, or unclear risk as implemented in RevMan 5.3 software.

### Data synthesis and meta-analysis

Statistical analyses of LVEF were performed using a comprehensive meta-analysis. Mathematical and computer modeling were also used to optimize management [23–25]. Data were calculated using the inverse variance formula. For continuous parameters, weighted mean differences (WMDs) were calculated using end of trial mean values, their corresponding standard deviations, and treatment arm size. Moreover, the data were analyzed using 95% confidence intervals (CI). A random-effect model was used. A forest plot was used to show the WMD and 95% CI for each study. The percentage of variability across studies that was attributable to heterogeneity beyond chance was estimated using *I* ^*2*^ and Q statistics. The Q statistic was considered significant if *P* < 0.05, and *I* ^*2*^ > 50% indicated high heterogeneity. Subgroup analyses were conducted based on transplantation time (<1 week or 1–4 weeks) after PCI and injected MSC dose (<10^7^ cells or >10^7^ cells). In studies reporting the mean ± standard error, the standard deviation of the required data was calculated using a previously used standardized formula [26]. A two-sided *P* value <0.05 was considered statistically significant. Statistical analyses were performed using Review Manager software (RevMan, version 5.3) and SPSS software (version 12).

### Sensitivity analyses and publication bias

Considering the small number of studies in this pooled analysis, the authors tested the robustness of our results in sensitivity analyses by omitting one study at a time. Potential publication bias was assessed using the Egger test and was represented graphically using Begg funnel plots, which are based on an adjusted rank correlation test.

## Results

### Study identification and selection

**Fig. 1:**
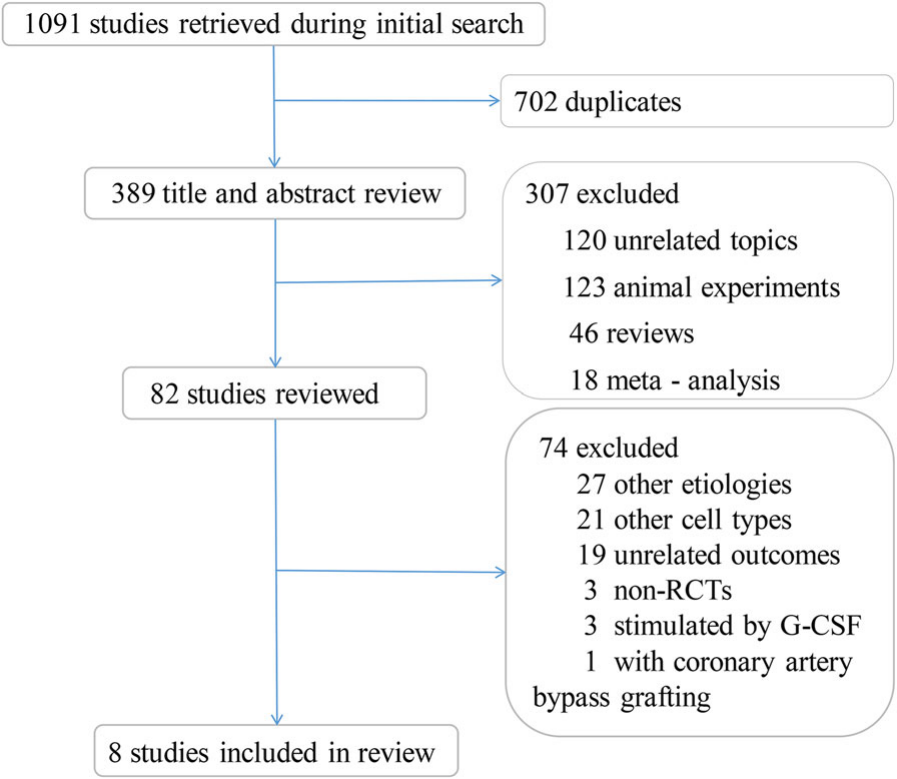
Flow diagram of enrolled trials. Flow diagram of randomized controlled trials (*RCTs*) selected for the meta-analysis of acute myocardial infarction with mesenchymal stromal cells therapy. *G-CSF* granulocyte colony-stimulating factor

A flow diagram depicting the overall search strategy is demonstrated in Fig. 1. Of 1091 articles retrieved during the initial search (371 from PubMed, 353 from MEDLINE, 342 from EMBASE, and 25 from the Cochrane Database), 702 were duplicates, 120 were excluded after title and abstract screening, 123 were animal experiments, 46 were reviews, and 18 were meta-analyses. The remaining 82 studies were retrieved in full for detailed evaluation. Twenty-seven were excluded due to etiology other than AMI, 21 used other cell types included with bone marrow stem cells, 19 were excluded due to unrelated outcomes, three were non-RCTs, three included G-CSF stimulation, one lacked a control group, and one included coronary artery bypass grafting. Eight RCTs with a total of 449 patients were eligible for review [7, 10, 12, 27–31].

### Study characteristics and study quality

The eight included studies involved patients with AMI, including patients with both ST-elevated and non-ST elevated myocardial infarction treated with primary PCI. The methodological quality of the enrolled studies was assessed using key indicators as shown in Table 1. The randomization methods used in the included trials were defined as being of low risk, except one which was reported as being at high risk of allocation concealment [30]. In addition, at least 50% (ranging from 50 to 75%) of the randomized patients were analyzed with regard to the outcomes of the binding assessment. Almost all the included trials were defined as being of low risk of incomplete outcome data and selective reporting. The main characteristics of the trials and patients are summarized in Tables 2 and 3. The trials were published between 2004 and 2015. The sample sizes ranged from 14 to 116, with follow-up ranging from 1 to 24 months. Of the eight trials that were identified, five were multicenter studies [10, 27–30]. The average participant age in the eight trials ranged from 48 to 59 years. Most participants were male. The mean age and gender were similar between the control and treatment groups within each study (*P* > 0.05). Most studies used a 1:1 randomization scheme. Seventy-five percent (6/8) of the trials used bone marrow-derived MSCs; one used MSCs from umbilical cord [27] and one used MSCs from adipose tissue [12]. One trial reported an initial LVEF of less than 45% [31], and two used intravenously injected MSCs [10, 29]. Considerable heterogeneity existed in the timing of cell transplantation after PCI (from within 24 h after transplantation to 25 days after) and in the number of cells administered (ranging from 3 × 10^6^ to 8 × 10^9^ cells).

**Table 1:** Quality assessment of included studies

Study	Random sequence generation (selection bias)	Allocation concealment (selection bias)	Blinding of participants and personnel (performance bias)	Binding of outcome assessment (detection bias)	Incomplete outcome data (attention bias)	Selective reporting (reporting bias)
Chen et al. 2004 [7]	L	L	U	U	L	L
Chullikana et al. 2015 [10]	L	L	L	L	L	L
Gao et al. 2013 [28]	L	L	L	H	L	L
Gao et al. 2015 [27]	L	L	L	L	L	L
Hare et al .2009 [29]	L	L	L	L	U	L
Houtgraaf et al. 2012 [12]	L	L	L	L	L	L
Lee et al. 2014 [30]	L	H	H	H	L	L
Wang et al. 2014 [31]	L	L	U	U	L	L

*H* high risk, *L* low risk, *U* unclear risk (Cochrane Handbook for Systematic Reviews of Interventions, Version 5.1.0)

**Table 2:** Characteristics of included trials

Study	Study design	Setting	Clinical scenario	Primary intervention	Sham infusion	Sample size, *n* (MSCs/control)	Endpoint	Mean follow-up time points (months)	Withdrawal number, *n* (%)
Chen et al. 2004 [7]	RCT	Single-center	AMI	PCI	Yes	69 (34/35)	LVEF, LVESV, LVEDV	3, 6	0 (0)
Chullikana et al. 2015 [10]	RCT	Multicenter	AMI	PCI	Yes	20(10/10)	LVEF, Infarct size, Perfusion defect	6, 24	4 (20)
Gao et al. 2013 [28]	RCT	Multicenter	AMI	PCI	Yes	43 (21/22)	LVEF, LVESV, LVEDV, WMSI, LVFS	6, 12, 24	4 (9)
Gao et al. 2015 [27]	RCT	Multicenter	AMI	PCI	Yes	116 (58/58)	LVEF, LVESV, LVEDV, WMSI, LVFS	1, 4, 12, 18	4 (3)
Hare et al. 2009 [29]	RCT	Multicenter	AMI	PCI	Yes	60 (39/21)	LVEF, LVESV, LVEDV	1, 2, 3, 6, 12	0 (0)
Houtgraaf et al. 2012 [12]	RCT	Single-center	AMI	PCI	No	14 (10/4)	LVEF, Infarct size, Perfusion defect	6	1 (7)
Lee et al. 2014 [30]	RCT	Multicenter	AMI	PCI	NR	69 (33/36)	LVEF, LVESV, LVEV, WMSI	1, 2, 6	11 (16)
Wang et al. 2014 [31]	RCT	Single-center	AMI	PCI	Yes	58 (28/30)	LVEF	1, 3, 6	3 (5)

*AMI* acute myocardial infarction, *LVEDV* left ventricular end-diastolic volume, *LVEF* left ventricular ejection fraction, *LVESV* left ventricular end-systolic volume, *LVFS* left ventricular fractional shortening, *MSC* mesenchymal stromal cell, *NR* not reported, *PCI* percutaneous coronary intervention, *RCT* randomized controlled trial, *WMSI* wall motion score index

**Table 3:** Patients and procedural characteristics of included trials

Study	Mean age (years)	Male (%)	Baseline LVEF (%) (mean ± SD)	Time from PCI to MSC therapy (days)	Cell resource	Mean cell dose	Delivery method
Chen et al. 2004 [7]	58	95.7	41.85 ± 8.3	18.3	BM	4.8–6 × 10^10b^	IC
Chullikana et al. 2015 [10]	48	90.0	43.25 ± 4.01	2	BM	2 × 10^6^	IV
Gao et al. 2013 [28]	57	93.0	50.71 ± 1.5	17.1	BM	3.08 × 10^6^	IC
Gao et al. 2015 [27]	57	94.6	51.51 ± 0.95	6.2	Umbilical cord	6 × 10^6^	IC
Hare et al. 2009 [29]	58	71.7	49.81 ± 10.25	<7	BM	80 × 10^6^	IV
Houtgraaf et al. 2012 [12]	59	78.6	45.36 ± 2.73	<1	Adipose tissue	1.74 × 10^6^	IC
Lee et al. 2014 [30]	54	75.4	49.57 ± 8.63	25	BM	72 × 10^6^	IC
Wang et al. 2014 [31]	57	60.3	28.70 ± 4.33^a^	15	BM	100 × 10^6b^	IC

^a^Highlights a considerable low baseline LVEF; ^b^highlights a significantly higher number of cells

*BM* bone marrow, *IC* intracoronary, *IV* intravascular, *LVEF* left ventricular ejection fraction, *PCI* percutaneous coronary intervention, *SD* standard deviation,

### Influence of transplantation time and cell dose on LVEF

**Fig. 2:**
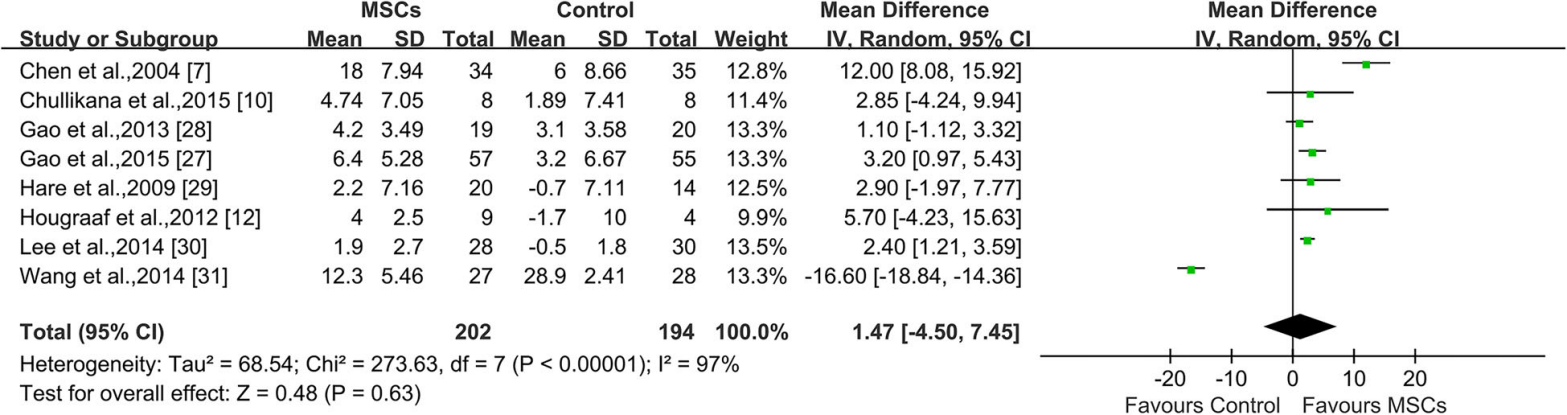
The effect of MSC therapy on left ventricular systolic function. Forest plot of weighted mean difference on LVEF compared with control. *CI* confidence interval, *IV* inverse variance, *MSC* mesenchymal stromal cell, *SD* standard deviation

The pooled comparison of the change in LVEF is shown in Fig. 2. The results revealed that LVEF was not statistically higher than baseline in patients in the MSC group compared with those in the control (1.47% increase, 95% CI −4.50 to 7.45; *P* = 0.63). A considerable degree of heterogeneity was observed (*I* ^*2*^ = 97%). Planned subgroup and sensitivity analyses were conducted to further explore the statistical heterogeneity.

### Subgroup analyses

#### Transplantation time

**Fig. 3:**
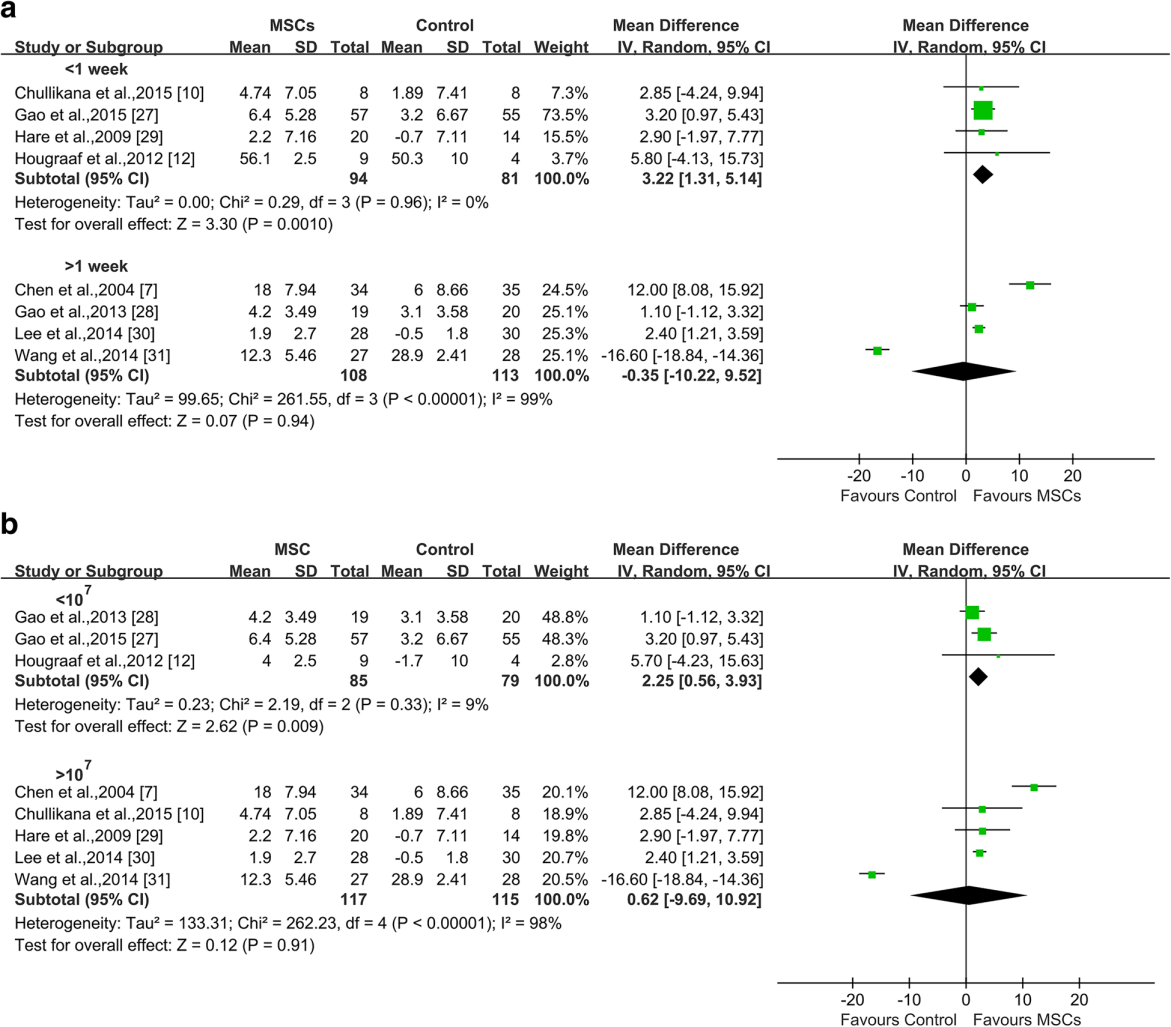
Impact of transplantation time and dose of MSCs on LVEF. **a** Forest plot showing the impact of transplantation timing of MSCs (<1 week or >1 week) on LVEF. **b** Forest plot showing the impact of transplantation dose of MSCs (<10^7^ cells or >10^7^ cells) on LVEF. *CI* confidence interval, *IV* inverse variance, *MSC* mesenchymal stromal cell, *SD* standard deviation

When comparing cell infusion time, greater LVEF improvement was observed in the groups with patients who were injected with MSCs within 1 week (3.22% increase in LVEF, 95% CI 1.31 to 5.14; *I* ^*2*^ = 0%; *P* = 0.001) compared with that in the control at 6 months of follow-up. However, the results were not statistically significant for groups involving the infusion of MSCs more than 1 week after PCI (−0.35%, 95% CI −10.22 to 9.52; *I* ^*2*^ = 99%; *P* = 0.94 compared with the control) (Fig. 3a).

#### Transplantation dose

In the trials involving an injected cell dose of less than 10^7^, LVEF was improved by 2.25% compared with the control (95% CI 0.56 to 3.93, *I* ^*2*^ = 9%; *P* = 0.009). However, MSCs at doses of greater than 10^7^ did not exhibit any LVEF benefit (0.62%, 95% CI −9.69 to 10.92; *I* ^*2*^ = 98%; *P* = 0.91) (Fig. 3b).

#### Combination of transplantation timing and dosage

**Fig. 4:**
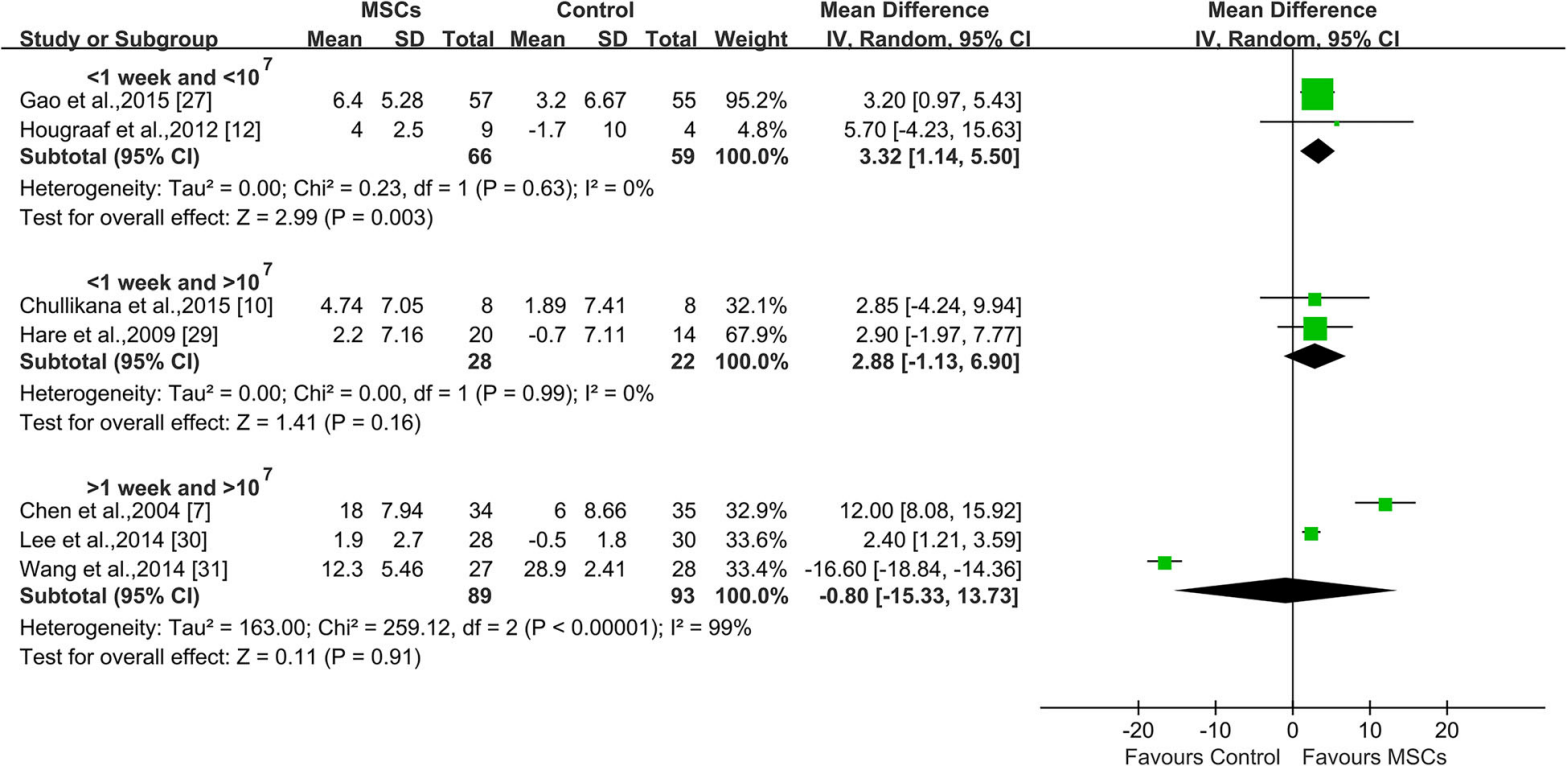
Impact of combining transplantation timing and dosage of MSCs on LVEF. Time and dose was divided into three subgroups: <10^7^ MSCs were injected within 1 week of AMI, >10^7^ MSCs were injected within 1 week of AMI, and >10^7^ MSCs were injected after 1 week of AMI. *CI* confidence interval, *IV* inverse variance, *MSC* mesenchymal stromal cell, *SD* standard deviation

Injection timing and the dose were also analyzed in combination (Fig. 4). LVEF improvements were only observed in the group in which MSCs were injected within 1 week and at a cell dose of less than 10^7^; this combination resulted in a significant increase in LVEF of 3.32% (95% CI 1.14 to 5.50; *I* ^*2*^ = 0%; *P* = 0.003); other transplantation timing and dose combinations showed no benefit in terms of LVEF (2.88%, 95% CI −1.13 to 6.90, *I* ^*2*^ = 0%; *P* = 0.16 for MSC injection within 1 week with a cell dose of greater than 10^7^ vs. control; -0.8%, 95% CI −15.33 to 13.73, *I* ^*2*^ = 99%; *P* = 0.91 for MSC injection within 1–4 weeks with a cell dose of greater than over 10^7^ vs. control).

### Publication bias and sensitivity analyses

The trial reported by Wang et al. [31] fell outside the Begg funnel plot, indicating publication bias. Using a sensitivity analysis, the same trial also exhibited heterogeneity. *I* ^2^ decreased from 95% to 75% and LVEF increased from 1.47% to 3.93% in the MSC group (*P* = 0.001 compared with the control) when the data from Wang et al. [31] were excluded.

## Discussion

The main result of this analysis is that MSC therapy might only achieve better LVEF improvement within a specific transplantation time window and when using an optimal MSC dose.

The efficacy of stem cell therapy in patients with AMI has long been discussed. Among the candidate cell types, MSCs are suggested to achieve better global LVEF, thus reducing infarct size and left ventricular remodeling compared with other cell types, such as CD34^+^/CD133^+^ and bone marrow mononuclear cells, in AMI [32, 33]. Interestingly, researchers are often uncertain about the timing and MSC dose that should be administered to achieve better LVEF in AMI patients. However, data addressing this issue are contradictory: Liu et al. reported that various doses of stem cells (hybrid cells) did not influence LVEF improvement in AMI [32], but other investigators have suggested that beneficial effects might only be achieved when higher numbers (>10^8^) of stem cells are infused [34, 35]. Thus, this meta-analysis addresses the optimal time window and dose of MSCs administered to AMI patients for the first time. No significant further LVEF increase was observed in the MSC group compared with the controls in all trials, and high heterogeneity (*I* ^*2*^ = 97%) was noted among the trials. The authors then explored whether the transplantation time and/or cell dose used explained the insignificant findings regarding LVEF.

### Effect of timing and MSC dose on LVEF

In the subanalysis of transplantation time, LVEF was increased by 3.22% in the MSC group compared with the controls when the cells were injected within 1 week after PCI. However, no benefit for LVEF over the controls was observed when MSCs were administered after more than 1 week. This finding is consistent with other randomized trials studying cell administration time using bone marrow stem cells that claimed that the best benefits for global LVEF were achieved when administering cell therapy between 4 and 7 days after AMI [36, 37]. Stem cells that are infused immediately after AMI might cause excessive obstruction and dysfunction in the microvascular bed, thereby creating a hostile environment due to inflammation of the myocardium potentially limiting cell retention and engraftment [38]. However, preclinical trials in which stem cells were administered within 1 week but not immediately indicated that administering stem cells served to prevent cardiomyocyte loss by secreting anti-apoptotic and anti-inflammatory paracrine factors or by inducing an angiogenic effect [39–41]. Moreover, the refusion time of MSCs was determined from PCI to cell administration, which is different from measuring the time interval starting at AMI. Considering that PCI is carried out as early as possible after AMI in clinical emergencies, the intracoronary or intravascular administration of stem cells may save viable myocardium after the reperfusion of infarcted myocardium. More clinical data and basic research are needed to address this issue.

Regarding the effects of cell dose on LVEF improvement, the results found in this study showed that an MSC dose of less than 10^7^ can provide a significant beneficial effect on LVEF, while higher doses (more than 10^7^) of MSCs exhibit no LVEF increase compared with the controls. However, several studies have reported that patients administered with a higher number (more than 10^8^ cells) of bone marrow stem cells exhibit a greater LVEF benefit [42, 43]. However, those who advocate using the standard number of infused cells have suggested that MSCs, which are larger (~22–25 μm in diameter) than capillaries (~8–10 μm in diameter), may be associated with a risk of obstructing microvessels and might compromise blood flow when injected intracoronarily [36, 44]. Gao et al. reported that one patient suffered from a serious complication involving coronary artery occlusion and subsequent lack of flow during the intracoronary procedure when injecting a higher dose (3.08 × 10^6^) of MSCs [28]. Vulliet et al. also reported the occurrence of coronary embolisms leading to acute myocardial ischemia and subacute myocardial microinfarctions after the intracoronary injection of MSCs in a dog model [45].

When studying injection timing and dose in combination, a significant impact on LVEF (an increase of 3.32%) was found in a subgroup combining a transplantation time within 1 week and an injected dose of less than 10^7^ cells (95% CI 1.14 to 5.50); transplanting the cells within 1 week and injecting more than 10^7^ cells resulted in a slight improvement of LVEF, whereas administering the cells at more than 1 week and injecting more than 10^7^ cells had the opposite effect on LVEF. However, these results should be cautiously interpreted as only two trials were included in this study, and one used a small patient samples. Notably, the data from Gao et al. markedly demonstrated a similar result with strong significance [27].

As we mentioned above, the preliminary results should be interpreted with caution. The potential of stem cell therapy for cardiac repair may be influenced not only by cell dosage but also by patient status, such as the level of basic ejection fraction (28.7 ± 4.33 to 51.51 ± 0.95 in the included studies). Moreover, the inter-study differences in patient age and gender may affect therapeutic effectiveness indicating that aging patients who are likely to suffer from impaired endothelium might exhibit inadequate physiological angiogenesis responses to ischemia and females may benefit more from stem cell therapy than males [46]. In addition, we suspect that the source and purity of MSCs can affect therapeutic response.

### Mechanisms of the therapeutic effect of MSCs

It has been widely demonstrated that the four main mechanisms of action for the cardioreparative effects of MSC therapy are as follows: (1) in vivo reduction of myocardial fibrosis; (2) stimulation of angiogenesis; (3) restoration of contractile function through engraftment differentiation; and (4) stimulation of endogenous cardiac stem cells to proliferate and differentiate [47]. Accumulating evidence has indicated that the paracrine mechanism is the predominant cause of the beneficial effects exerted by MSCs and is based on a multitude of bioactive molecules, including cytokines, chemokines, and growth factors [48]. These molecules contribute to reducing fibrosis through suppressing the proliferation of fibroblasts and promoting their metalloproteinase secretion, stimulating the angiogenesis, proliferation and differentiation of host cells, and recruiting endogenous cardiac stem cells. In addition, MSCs can alter endothelial cell behavior and differentiate into endothelial cells in vitro or into cardiomyocytes in vivo. Moreover, implanted MSCs can regulate the proliferation and differentiation of endogenous cardiac stem cells and enhance myocyte cell cycling via cell–cell interaction, thereby homing to the injury site to repair injured myocardium and boost angiogenesis for myocardial repair [49]. In addition, it has been reported that direct cellular mechanisms involving exosomes, mitochondrial transfer, connexin43, etc., can convincingly explain the effects observed in preclinical and clinical studies [47]. Moreover, studies also indicate that the mesenchymal–endothelial transition appears to have an important physiological role in cardiac repair due to possible neovascularization [50].

### Limitations

This study has several limitations. First, the scope of the results is limited by the small number of trials included. Ideally, more observation parameters regarding endpoints, such as LVESV, LVEDV, and the wall motion score index, could be included to evaluate cardiac structure and function when more RCTs are conducted. Second, as the authors mentioned previously, the results describing the combination of cell injection timing and dose were drawn from only two trials; this might reduce the power of the study to draw solid conclusions. However, a separately conducted meta-analysis on timing and dose including more trials is expected to draw the same conclusions. Finally, a trial from Wang et al. applied MSCs in patients with severely impaired LVEF (<35%), and this might have affected the inhomogeneity of the meta-analyses. Detailed conclusions regarding baseline LVEF and LVEF changes during follow-up require support from more data.

## Conclusion

Transplantation time and injected cell dose are key factors that determine the therapeutic effect of stem cell therapy. No more than 10^7^ MSCs injected within 1 week for AMI after PCI might represent the optimal time window and dose for improving left ventricular systolic function; these results will hopefully provide a reference for future research and clinical studies.

## Abbreviations

AMI: Acute myocardial infarction
CI: Confidence interval
G-CSF: Granulocyte colony-stimulating factor
LVEDV: Left ventricular end-diastolic volume
LVEF: Left ventricular ejection fraction
LVESV: Left ventricular end-systolic volume
MRI: Magnetic resonance imaging
MSC: Mesenchymal stromal cell
PCI: Percutaneous coronary intervention
RCT: Randomized controlled trial
WMD: Weighted mean difference
